# New Insights into the Mechanical Behavior of Thin-Film Composite Polymeric Membranes

**DOI:** 10.3390/polym14214657

**Published:** 2022-11-01

**Authors:** Fatima Ghassan Alabtah, Abedalkader Alkhouzaam, Marwan Khraisheh

**Affiliations:** Mechanical Engineering Program, Texas A&M University at Qatar, Doha 23874, Qatar

**Keywords:** thin-film composites, polymeric membranes, mechanical behavior, layer-by-layer analysis

## Abstract

Limited predictions of thin-film composite (TFC) membranes’ behavior and functional life exist due to the lack of accurate data on their mechanical behavior under different operational conditions. A comprehensive investigation of the mechanical behavior of TFC membranes addressing deformation and failure, temperature and strain rate sensitivity, and anisotropy is presented. Tensile tests were conducted on commercial membranes as well as on individual membrane layers prepared in our laboratories. The results reveal the overall mechanical strength of the membrane is provided by the polyester layer (bottom layer), while the rupture stress for the middle and top layers is at least 10 times smaller than that of the polyester layer. High anisotropic behavior was observed and is attributed to the nonwoven structure of the polyester layer. Rupture stress in the transverse (90°) direction was one-third of the rupture stress in the casting direction. Limited temperature and strain rate dependence was observed in the temperature range that exists during operation. Scanning electron microscopy images of the fractured surfaces were also analyzed and correlated with the mechanical behavior. The presented results provide new insights into the mechanical behavior of thin-film composite membranes and can be used to inform novel membrane designs and fabrication techniques.

## 1. Introduction

Water demand rises in tandem with population increase, economic development, and evolving consumption habits [1]. Over the last century, global water demand has surged by 600 percent. This equates to a 1.8 percent yearly growth rate [2]. As a recently developed technology, desalination has become a credible, more efficient, and cost-competitive alternative water source in response to increasing water constraints. Utilizing semi-permeable seawater reverse osmosis (SWRO) membranes has dominated the desalination industry over the past ten years [3]. Water treatment membranes must be durable and mechanically strong to deliver high flux and contaminant rejection competences. RO membranes are usually operated under harsh conditions and being subjected to high pressures and complex stresses. Thus, it is critical to evaluate the mechanical characteristics of those membranes to explore the failure processes, such as surface damage, chemical and mechanical degradation, delamination, and lack of membrane stability under different conditions. The majority of published research was concerned with the desalination of saltwater or membranes for wastewater treatment, with emphasis on surface modification [4,5,6], membrane processing [7,8,9], and antifouling characteristics [10,11,12]. According to the Scopus database, the number of papers published each year on the mechanical properties of thin-film composite (TFC) membranes still only accounts for 5% of the yearly publications on water membranes [13].

As reported in the literature, the water membrane’s functionality is greatly influenced by the mechanical stress applied during filtration, cleaning, and different operating conditions. The integrity and reliability of membranes counts on their surface physical characteristics, which degrade when subjected to different loading conditions. Aghajani et al. [14] reported the correlation between permeability and deformation for ultrafiltration (UF) and microfiltration (MF) membranes, and it was revealed that the permeability of the MF and UF membranes drop as compressive strain rises. Idarraga-Mora et al. [15] examined the impact of woven polyester mesh size on the mass-transfer resistance and bursting strength of TFC membranes used for pressure-retarded osmosis (PRO) process. The results demonstrated that TFC membranes prepared with woven backings withstand loadings similar to those experienced by commercial SWRO membranes. It has also been found that woven mesh polyester backings offer the overall PRO structure substantial strength; however, the porous support layer experiences deformations that rely on the backing’s opening size. Then, in 2020, the same research group [16] studied the effect of mechanical strain on the behavior of commercial TFC membranes; they found out that, after exposure to linear strain values, the membranes’ transport characteristics were slightly altered. In addition, they found that higher salt transport through membranes would ensue due to local distortion and defect. Accordingly, it was evident from the literature that the membranes used in water treatment and desalination are reliable and durable mainly due to their mechanical strength. Reduced membrane stiffness and strength may result from mechanical degradation brought on by fouling, physical harm, physical pore plugging, backflow, and chemical treatment [17]. In addition, this mechanical degradation will significantly impact the permeability, particle rejection, and permeate quality [18].

Our previous publication [19] observed anisotropic behavior when the cruciform specimens were tested under biaxial tensile loading. The transverse direction was shown to have significantly greater strength than the longitudinal direction since TFC membranes are asymmetric (anisotropic) membranes composed of different layered materials determined by the species to be separated and their nature, where the thin separating layer is where most of the flow resistance is generated [20]. The intermediate porous layer does not influence the transport resistance of the permeate along the membrane; typically, the mechanical strength needed, as well as other elements such as chemical resistance and durability, are taken into consideration when choosing the material for the support layer. The complexity of TFC membrane behavior under varied loading circumstances was underlined in our previous study. The perceived anisotropic behavior revealed the membrane’s non-uniform properties, demonstrating that TFC membranes are chemically and structurally heterogeneous. To this end, it is critical to understand the mechanical characteristics of water treatment membranes and the deformation mechanisms to enhance the membrane structure design and accurately predict membrane failures. As an extension to our previous work, this work presents a new insight and foundational understanding of the mechanical properties of commercial TFC-RO membranes, first by investigating their strain rate sensitivity under different conditions and temperatures, where uniaxial tensile experiments have been conducted at dry and wet conditions at two different temperatures (22 °C and 40 °C) and different strain rates (0.01 s^−1^, 0.003 s^−1,^ and 0.0001 s^−1^). Second, RO membrane, polyester, polysulfone (PSF), and polysulfone + polyamide (PSF-PA) membrane layers prepared in our laboratories were tested at different orientations with respect to the casting direction (i.e., 0°, 45°, and 90°) to identify the layer that is dominating the anisotropic behavior and to detect the anisotropy effect on the stress–strain behavior. The tests were conducted using uniaxial testing equipment with a custom-designed temperature-controlled bath. Engineering stress–strain curves of the different tested samples were presented, compared, and discussed. Failure modes and fracture morphologies were observed by scanning electron microscopy (SEM).

It is worth highlighting the importance of understanding the membranes’ behavior under these different conditions. In addition to the fact that these results and information do not exist in the open literature, they are closely related to the real operational conditions:

- Temperature: The major performance indicators for a reverse osmosis unit are the flow rate of the produced fresh water and salt rejection. The feed water temperature has a significant impact on them [21]. The maximum allowable temperature range, according to the RO membrane manufacturers, is usually between 35 and 45 °C. It is for this reason that the membranes in this study were tested at 22 °C and 40 °C.
- Strain rate: The membrane may be subjected to non-uniform mechanical loading during casting, installation, handling, and operation. In addition, as membranes foul, the module will exhibit pressure variation resulting in a non-uniform loading rate. Not to mention the chemical cleaning that is performed at different flow rates and directions than the feed water. Therefore, it is critical to understand the behavior of the membranes under different loading rates.
- Anisotropy: TFC membranes are asymmetric (anisotropic) membranes made of several layered materials, where the majority of the flow resistance is obtained in the top thin separating layer. They have a complex behavior when subjected to varied loading conditions, in addition to the non-uniform properties, demonstrating that TFC membranes are chemically and structurally heterogeneous. Therefore, in order to improve the design of membrane structures and accurately predict membrane failures, comprehensive failure analysis in different directions is needed. It is also important to understand the layer-by-layer mechanical properties of RO membranes and their deformation mechanisms.
- Wet/Dry conditions: Thin-film membranes are manufactured in dry conditions and subjected to mechanical loading during installation and handling while still in their dry condition. However, in order to simulate the real operating conditions, the membranes should be tested while immersed in water (wet condition).

The novelty of the present study is that, for the first time, it comprehensively addresses the mechanical behavior of TFC membranes, including deformation and failure, strain rate sensitivity, anisotropy, and temperature dependence. The presented data can serve as guidelines for the future design of novel water treatment membranes for pressurized osmotic processes.

## 2. Materials and Methods

### 2.1. Materials

**Figure 1:**
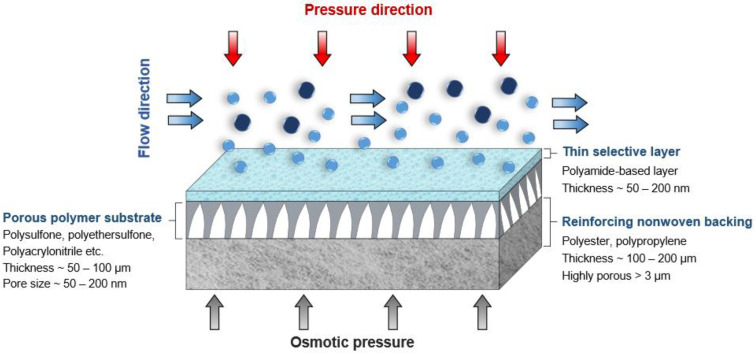
Schematic for the structure of the TFC membranes.

A commercial RO membrane (BW30-LE, FilmTec^TM^) supplied by DuPont, Wilmington, Delaware, USA, was used as a model polyamide-TFC (PA-TFC) membrane for mechanical testing. TFC membranes are made up of three layers: a reinforcement layer (nonwoven polyester) to offer structural support for high-pressure operations, a porous support layer (PSF), and a thin polyamide (PA) layer for separation control (Figure 1). 1-Methyl-2-pyrrolidinone (NMP, 99.5%) was obtained from Honeywell Chemicals, Muskegon, Michigan USA. Polysulfone (PSF, average Mw ~35,000), m-Phenylenediamine (MPD), and Polyvinylpyrrolidone powder (PVP, average Mw ~29,000) were obtained from Sigma Aldrich, Missouri, USA. 1,3,5-Benzenetricarbonyl Trichloride (TMC, >98%) was purchased from TCI America, Portland, Oregon, USA. N-hexane (99%) was purchased from SD Fine Chem Limited, Gujarat, India. Deionized water (DIW) was produced using the PURELAB Flex system Elga, Illinois, USA. All chemicals were used as purchased without further purification.

#### 2.1.1. Polysulfone (PSF) Membrane Preparation

A free-standing PSF membrane (backing-free) was fabricated to eliminate the effect of polyester fabric on the mechanical properties of TFC membranes. The PSF substrate was fabricated via the non-solvent-induced phase separation (NIPS) approach (commonly known as phase inversion), described elsewhere in the literature [22]. Briefly, a solution consisting of 17 wt.% PSF, 3 wt.% PVP and 80 wt.% NMP was used as the main dope solution. PSF was used as the main polymer matrix, while PVP was used as a pore-forming agent. The dope solution was stirred vigorously at 500 rpm at 60 °C for 3 h, followed by slower stirring at 130 rpm at room temperature overnight to form a transparent homogenous solution. The dope solution was then coated on a clean glass plate at a uniform speed and thickness. The casting speed was controlled using an automated coating machine (LR-G011 Automatic bar coater, Changsha Lonroy Technology Co. Ltd., Changsha, China). Two PSF membranes at two different thicknesses (20 and 30 µ) were cast using a film applicator (Elcometer 3580, Elcometer^®^, Manchester, UK). The casted membranes were then immersed in a coagulant bath (DIW), where the phase separation occurred. To ensure an ideal phase separation, the membranes underwent several washings and were kept in DIW overnight. The resulting membrane thickness is usually higher than the casting thickness as the membrane tends to swell and form a sponge-like structure throughout the phase inversion process. The final membrane thickness was estimated using scanning electron microscopy (SEM).

#### 2.1.2. Preparation of Backing-Free TFC Membranes

**Figure 2:**
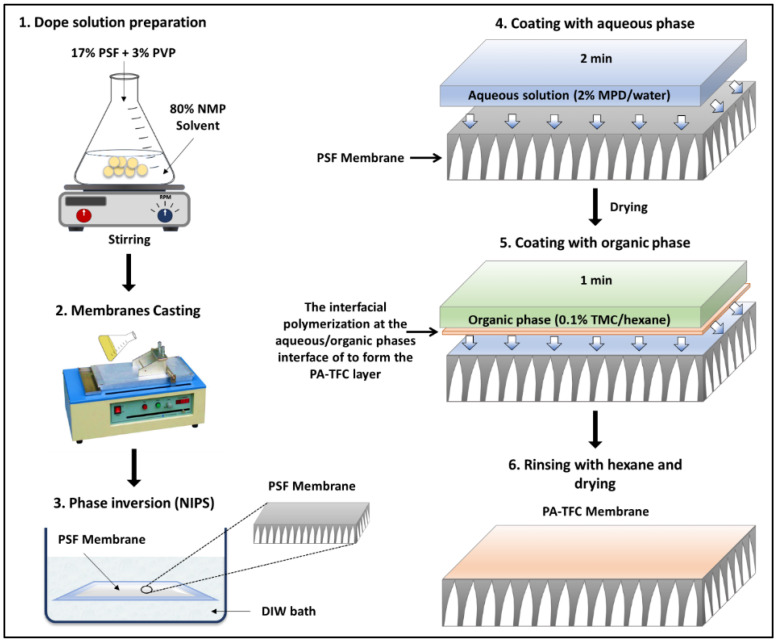
Schematic illustration for the phase inversion and interfacial polymerization approaches used for the TFC membranes fabrication.

The nonwoven backing-free TFC membranes were prepared using the interfacial polymerization technique. Interfacial polymerization is one of the renowned approaches for fabricating PA-TFC membranes [23]. This approach depends on the polymerization reaction of two monomers dispersed into two immiscible solvents (organic/aqueous), where the PA layer is formed at the interface of the organic/aqueous phases [24]. In brief, the prepared PSF substrates were fixed and clamped between two acrylic frames. Then, 2% MPD in water and 0.1% TMC in hexane were used as the aqueous and organic solutions. The MPD aqueous solution was poured and allowed to contact the membrane surface for 2 min. The excess solution was poured off, and the membrane dried at room temperature. The TMC organic solution was poured and contacted the membrane surface for 1 min. The coated membranes were then rinsed with hexane to remove the unreacted species, dried at room temperature, and finally stored in DIW overnight. Figure 2 illustrates the fabrication process of the backing-free PSF and TFC membranes investigated in this study. The description of the fabricated membrane layers, in addition to their SEM cross-sectional images, are shown in Table 1, while Table 2 presents schematic illustrations of the different tested membranes.

### 2.2. Tensile Samples Preparation and Testing

**Figure 3:**
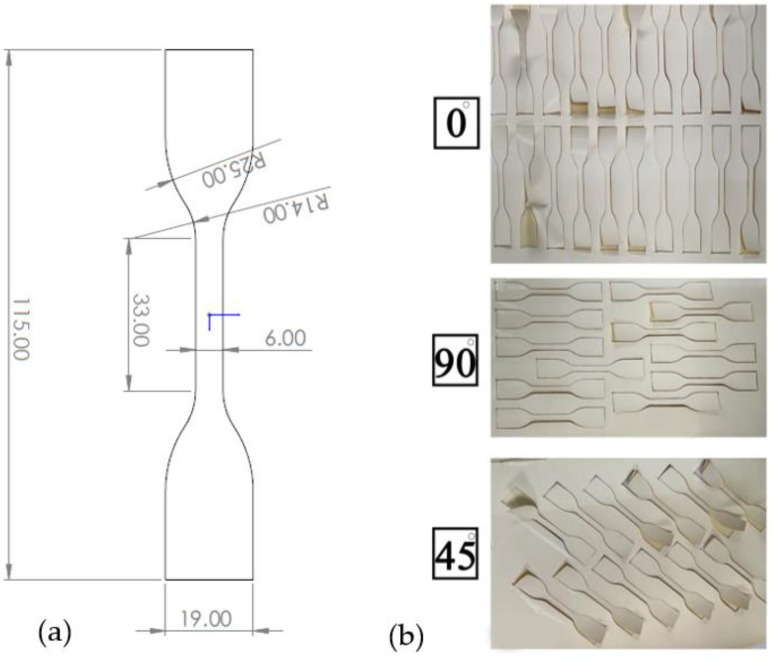
Tensile sample: (**a**) dimensions (mm) and (**b**) different cutting angle orientations.

The tensile test specimen was conformed to the dimensions shown in Figure 3a according to the D638-14 ASTM standard. A coherent METABEAM 400 laser cutting machine was used to cut the samples at a power of 100 watts and a cutting speed of 845 mm/s. The BW30, Polyester, PSF, and backing-free TFC membrane specimens were cut at three different orientations (0° (along with rolling direction), 45°, and 90°) as shown in Figure 3b. To avoid any unintentional scratches or contamination, the membrane samples were carefully rinsed with deionized water and allowed to dry overnight at room temperature in a clean area.

In this investigation, a ZwickRoell customized uniaxial testing equipment was used to conduct the tensile testing, which is specifically intended to be employed in testing thin films. The electromechanical actuators provide a maximum displacement of 200 mm for each axis and a maximum speed of 10 mm/s. One-sided closing screw grips with 300 N F_max_ are included with each actuator, in addition to a 2 kN load cell designed to measure static and dynamic tensile and compression forces. A laserXtens/VideoXtens compact device from Messphysik is mounted on the top, with one uEye camera and an f = 75 mm objective lens, with an attached adjustable LED incident light for the illumination of the specimen. The videoXtens mode was used in this research, where the system measured the displacement of two marks (targets) on the specimen, either dry or through the liquid. The extensometer system provides a signal for each testControl unit corresponding to the axial displacement controlled by this axis, allowing strain control. A Digital/Analog converter and sensors feed these signals into the testControl unit electronics. The measuring electronics for the force measurement complies with ISO 7500-1, ASTM E4.

**Figure 4:**
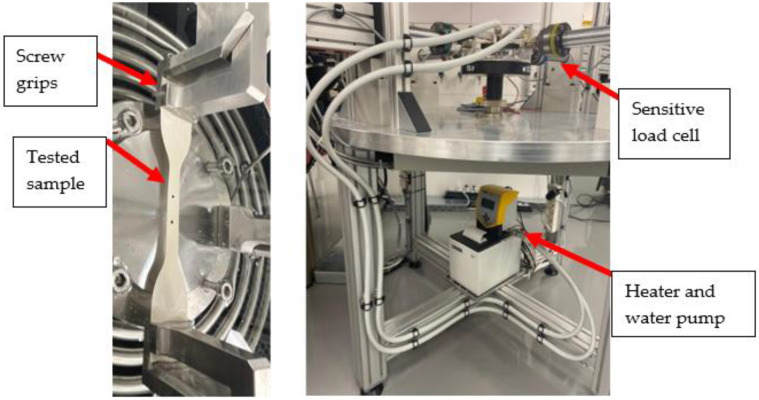
Bath heater and thermoregulation bath.

In order to investigate the stress–strain behavior of the commercial thin-film composite (TFC) membranes under different conditions and temperatures, uniaxial tensile experiments were conducted in dry and wet (immersed in a water path during testing) conditions at two different temperatures (22 °C and 40 °C), and different strain rates (0.01 s^−1^, 0.003 s^−1^, and 0.0001 s^−1^). Additionally, the specimens that were extracted from different orientations (0°, 45°, and 90°) were tested to detect the anisotropy effect on the stress–strain behavior. A plexiglass conditioning vessel thermoregulation bath was used to test the samples in a wet environment and under elevated temperatures (Figure 4). The thermoregulation bath was centrally mounted on the baseplate, where the connector is screwed onto the testing system’s base crosshead and can be adjusted to the required height by turning the knurled nut. Additionally, to control the temperature of the liquid in the bath, it was connected to a bath heater with a temperature range of 20 to 80 °C (Figure 4). Fracture morphologies after tensile tests were imaged utilizing SEM.

## 3. Results and Discussion

### 3.1. Effect of Membrane Orientation on Tensile Behavior

**Figure 5:**
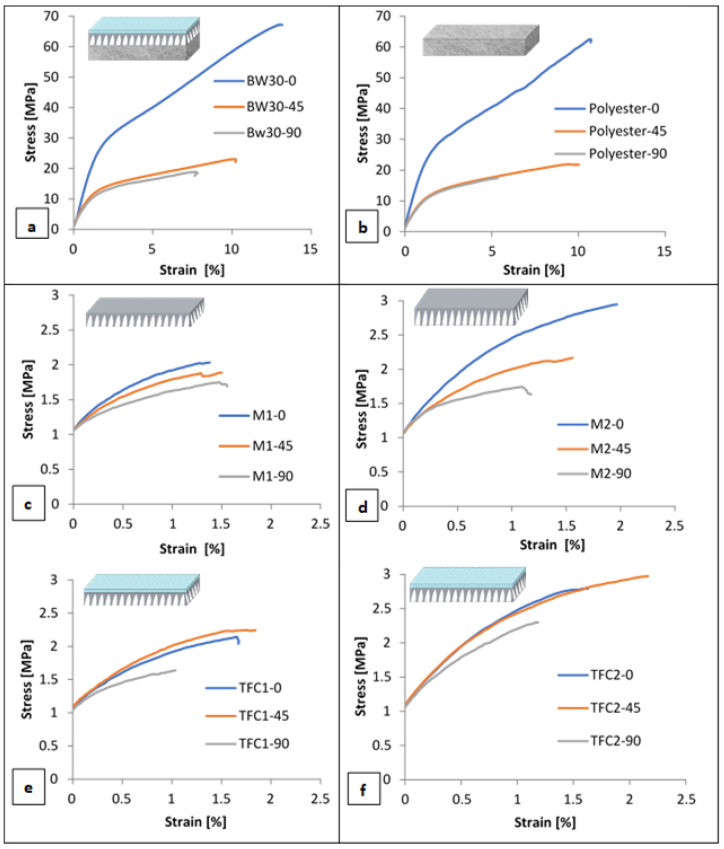
Engineering stress versus strain curves of 0°, 45°, and 90°: (**a**) BW30, (**b**) polyester, (**c**) M1, (**d**) M2, and (**e**) TFC1 and (**f**) TFC2 specimens.

**Figure 6:**
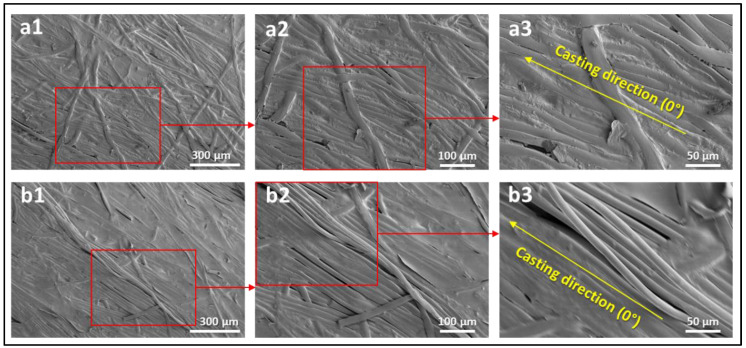
SEM images of (**a1**–**a3**) unloaded polyester and (**b1**–**b3**) loaded polyester near the fracture region.

Figure 5 presents the engineering stress–strain curves of samples cut at 0°, 45°, and 90° with respect to the casting direction for BW30, Polyester, M1, M2, TFC1, and TFC2 membrane samples. The uniaxial tensile testing for all conditions was carried out for three identical specimens, and the difference in the stress level among repeated tests for identical specimens was not exceeding 5%. The 0° specimens exhibited the highest peak stress in all types of specimens (BW30, polyester, and polysulfone (PSF) membranes). The rupture stress for the 0° direction in BW30 and polyester samples was approximately three times higher than in the other directions. This can be attributed to the higher number of polyester fibers oriented in the casting direction (0° direction), resulting in a higher tensile strength. This can be further confirmed by the SEM images presented in Figure 6 of the loaded and unloaded polyester samples. Hence, this direction produces higher interfiber frictional forces, which prevents breakage. At the same time, the 90° specimens displayed the lowest peak stress in all the cases. The differences in the stress levels of the different PSF and backing free-TFC membranes at different orientations are minimal and can be neglected when compared to the differences in the case of polyester and polyester-supported samples; thus, it could be concluded that the polyester layer dominated the anisotropic behavior of the entire RO membrane, due to the fact that it has a nonwoven structure (Figure 6). These findings match the results presented by Chauhan et al. [25], Pramanick et al. [26], and Ray et al. [27]. They assessed the nature of anisotropy in terms of tensile properties for nonwoven fabrics. It was found that the tensile strength in the machine direction (casting direction) is generally more than that in the cross direction. It can also be noticed that the backing-free PSF and TFC membranes exhibit the same stress–strain behavior. This can be attributed to the extremely low thickness and fragility of the PA layer that led to an unnoticeable influence on the mechanical properties.

Table 3 summarizes the mechanical properties of the different tested 0° membranes under dry (22 °C) conditions. By analyzing the data in the table, it is clear that the values for the entire membrane are close to the values of the polyester layer, demonstrating that the overall mechanical behavior of the membrane is determined by the polyester layer. It is also evident that there is a mismatch between the mechanical properties of the different layers that may lead to different types of failures within the layers. Surface delamination and cracks may develop on the top thin layers at loads way below the “expected” failure loads of the entire membrane. The presented data provide new insight into the mechanical performance of each layer within the thin-film composite membranes and help in developing a better understanding of their behavior and functional life during operation. It can also inform novel membrane designs with customized properties for the individual layers that will minimize the mismatch of mechanical properties for improved durability and integrity of membranes. In addition, these data explain why some membranes undergo unexpected failures during operation.

### 3.2. Strain Rate Sensitivity and Effect of Temperature on Tensile Behavior

**Figure 7:**
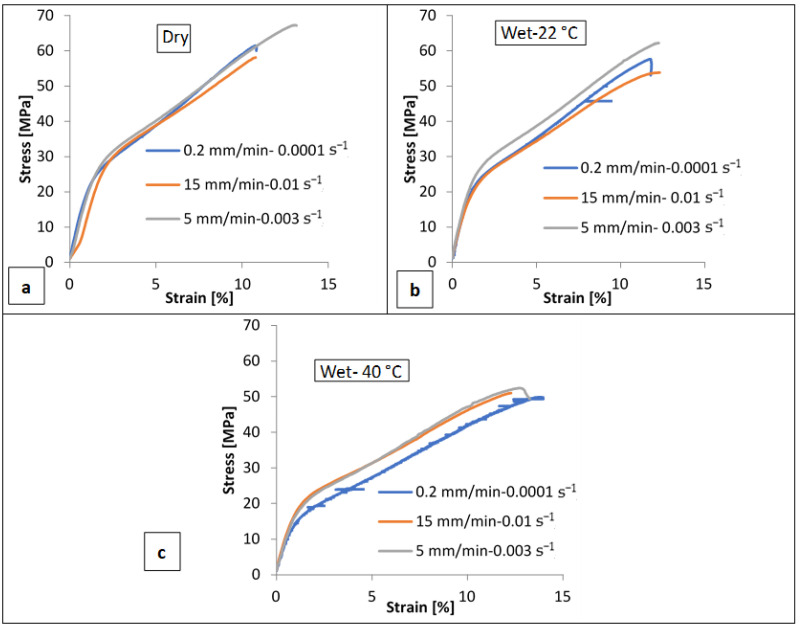
Engineering stress versus strain curves of BW30-0° specimens at 0.01 s^−1^, 0.003 s^−1^, and 0.0001 s^−1^ strain rates under (**a**) dry, (**b**) wet-22 °C, and (**c**) wet-40 °C conditions.

Figure 7 presents the engineering stress–strain curves for three sets of BW30-0° membrane specimens under dry, wet-22 °C, and wet-40 °C conditions. The uniaxial tensile tests for each set of specimens were conducted at the same temperature using three different strain rates (0.01 s^−1^, 0.003 s^−1^, and 0.0001 s^−1^) determined by the speed of movement of the tensile tester’s crosshead. It could be noticed that, when the samples were tested at a constant strain rate, the stress increased linearly and quickly during the first few seconds, the increases started to become non-linear, and an inflection point can be seen to have occurred around 2% strain, where the rate of increase gradually slowed down until it reached the point of fracture. No increase in the membranes’ strength can be highlighted with the increasing strain rate in the cases of dry and wet-22 °C conditions. It can be clearly noticed that BW30 membranes under the conditions mentioned above have limited strain rate sensitivity. While for the wet-40 °C condition, at 0.0001 s^−1^ strain rate, the material’s modulus (determined by the material’s initial slope of the tensile stress–strain response) and yield strength (maximum stress that was developed in the material without causing plastic deformation) are both at their lowest points. As a result of the increased strain rate, the material appears to be slightly stronger and stiffer. Generally, polyester fibers are largely dependent on intermolecular forces among its chains. As the temperature increases, polyester fibers may undergo softening, which will affect the intermolecular forces. Moreover, it is well known that strain rate sensitivity becomes more visible at elevated temperatures.

**Figure 8:**
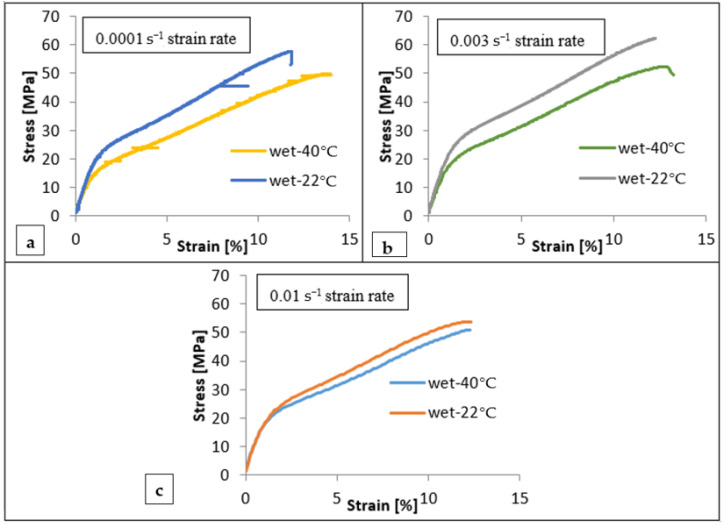
The effect of temperature on the engineering stress versus strain curves of the BW30-0° specimens at (**a**) 0.0001 s^−1^, (**b**) 0.003 s^−1^, and (**c**) 0.01 s^−1^ strain rates.

Figure 8 presents the effect of the temperature on the engineering stress–strain curves of BW30 specimens at different strain rates. When comparing the results in Figure 8, the increased temperature can be seen to result in slightly lower flow stress with no significant change to ductility. This result is in agreement with the findings obtained by Clerc et al. [28], who presented an investigation of the mechanical behavior of polyester fibers over a range of temperatures, and their results revealed that, as the temperature increased, the tensile strength and elastic modulus gradually decreased. In addition, failure strain increased from 14% to more than 40% with increasing temperature from 20 to 180 °C due to fiber softening. In contrast, the behavior will completely change when the temperature exceeds the melting point of the polyester. Choi et al. [29] revealed that the increase in temperature initiates a thermal bonding between polyester fibers, and the void content in the nonwoven fabrics would decrease, leading to a high thermal shrinkage and a sharp increase in tensile strength and modulus of the nonwoven fabrics when treated at a higher temperature than the melting temperature, as a result of fabric hardening and increase in density of nonwoven fabrics. Table 4 summarizes the mechanical properties of the different tested BW30-0° membranes under wet (22 °C and 40 °C) conditions and different strain rates.

### 3.3. Surface Characterization and Failure Modes

**Figure 9:**
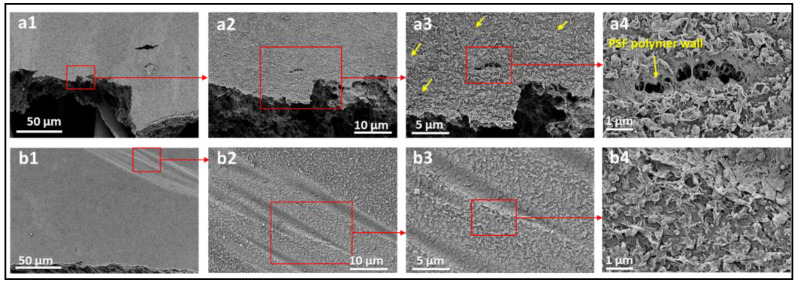
SEM images of BW30-0° membrane tested under (**a1**–**a4**) dry condition and (**b1**–**b4**) wet condition. The small yellow arrows indicate the regions of PA layer delamination in dry conditions.

**Figure 10:**
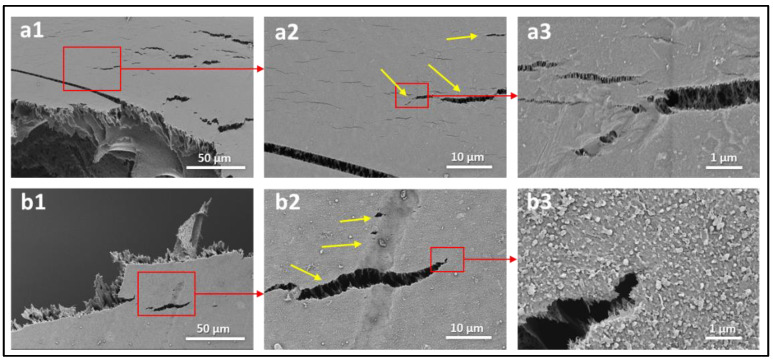
SEM images of the backing-free (**a1**–**a3**) PSF (M2) and (**b1**–**b3**) TFC2-0° membranes tested under dry conditions. The yellow arrows indicate cracks observed in areas close to the fracture region.

To evaluate and understand the failure modes of each layer of the tested membranes, scanning electron microscopy (SEM) images were obtained at areas near the fracture region of each membrane. The SEM analysis was performed using the Apero-S device manufactured by Thermo Fisher Scientific. All membrane samples were washed multiple times with distilled water to eliminate contamination or inadvertent scratches and left to dry in a sterile environment. The samples were coated with platinum to improve their conductivity before SEM examination. Figure 9 shows the SEM images of the commercial BW30 membrane tested under dry and wet conditions near the fracture region. The results show some cracks and deformations in the top layers (PSF-PA) when tested under dry conditions, as depicted by the high magnification images in Figure 9(a3,a4). The yellow arrows indicate different regions of these deformations, and the damage extends to the middle layer (PSF), as shown in Figure 9(a4). Wet membranes exhibited different behavior where no obvious cracks were observed, even in areas near the fracture region. Additionally, the morphological change in wet conditions was in the form of stretches and wrinkles, as shown in Figure 9(b1–b4). High magnification images of the wet membranes (Figure 9(b3,b4)) suggest that the top layers remain coherent even in areas near the fracture region. This can be explained by the fact that PSF is brittle in dry conditions and has higher ductility in wet conditions [17]. These findings are supported by the SEM images of the backing-free membranes tested under the dry conditions shown in Figure 10. The widespread cracks observed on M2 and TFC2 surfaces indicate a brittle failure of these membranes in dry conditions. The findings of Figure 9 and Figure 10 imply that the top layers are more susceptible to deformation at much lower loading levels than the stress-fracture levels in dry conditions. Contrastingly, in wet conditions, these layers have higher ductility than the backing polyester layer, which results in some stretches and wrinkles in the top layers while remaining cohesive.

## 4. Conclusions

We presented comprehensive mechanical testing, supported by scanning electron microscopy, of a thin-film composite (TFC) RO membrane and its individual layers under various conditions. It was shown that the overall mechanical behavior of the TFC membrane is dominated by the polyester backing layer, which also causes the high anisotropic behavior. The layer-by-layer analysis revealed a significant mismatch between the mechanical properties of the middle and top layers compared to the polyester backing layer, which may lead to surface delamination and cracks at lower-than-expected loads. Another important result was observed for samples tested under wet conditions. The middle and top layers exhibited more ductile behavior compared to dry conditions, as was demonstrated by the wrinkles and lack of cracks. This can be attributed to the stability of the top layer under wet conditions. For the temperature range studied in this work, the membranes exhibited a limited strain and temperature dependence. A slight softening effect on the stress level was observed at 40 °C.

Although this study presented new data on the mechanical and failure behavior of TFC RO membranes that can help in the design and fabrication of novel membranes, it also identified areas that need further investigation. Comprehensive biaxial testing under static and fatigue loading is needed to understand how these membranes behave under real conditions. Additionally, attention should be placed to the interfacial properties between the various layers. It is hoped that this study will encourage more mechanical investigations of TFC RO membranes, as the available data are very limited.

## Figures and Tables

**Table 1:** PSF and TFC prepared membrane layers used in this work.

Membrane	Description	Membrane Thickness (µm) *	Cross-Sectional SEM
M1	PSF membranes casted at thickness of 20 µm	129.7 ± 6.7	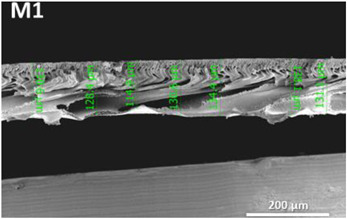
M2	PSF membranes casted at thickness of 30 µm	201.3 ± 5.4	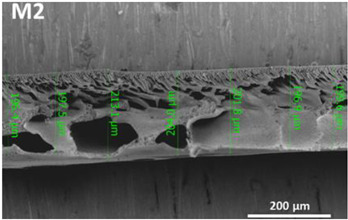
TFC1	PA-TFC membranes fabricated on M1 membrane	128.1 ± 2.7	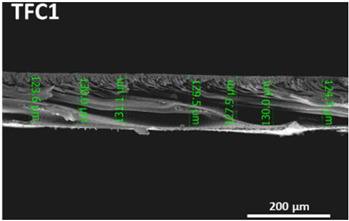
TFC2	PA-TFC membranes fabricated on M2 membrane	204.5 ± 4.4	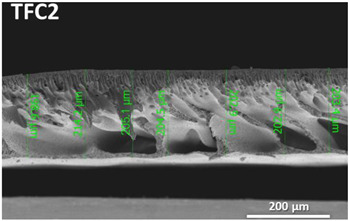

* Membrane thickness was estimated using cross-sectional SEM images at seven different points. The (±) sign indicates the standard deviation of the estimated points.

**Table 2:** Schematic illustration of the types of the tested membranes.

Sample	Schematic Illustration
BW30–commercial RO membrane	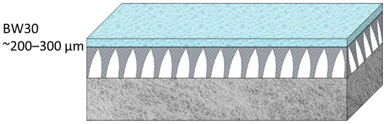
Polyester layer	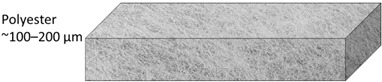
M1	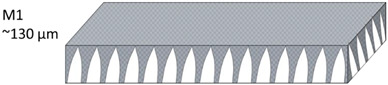
M2	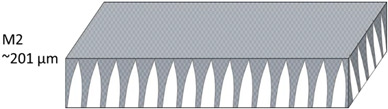
TFC1	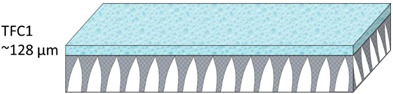
TFC2	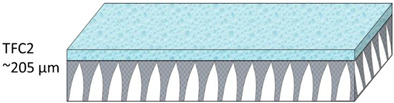

**Table 3:** Mechanical properties of the different tested 0° membranes under dry (22 °C) condition.

Sample	Modulus of Elasticity (E) in (MPa)	Flow Stress (Yield Strength) in (MPa)	Ultimate Strength in (MPa)	Rupture Stress in (MPa)	Rupture Strain in (%)
BW30	18.786	20.323	67.25028	67.1514	13.16729
Polyester	17.433	18.736	62.51793	61.5823	10.69662
M1	1.465	1.159	2.036885	2.027712	1.338402
M2	2.242	1.475	3.14976	2.900595	2.541783
TFC1	1.361	1.305	2.146464	2.040404	1.670021
TFC2	2.152	1.401	2.796622	2.68501	1.58337

**Table 4:** Mechanical properties of BW30 membranes under wet (22 °C and 40 °C) conditions and different strain rates.

Sample	Modulus of Elasticity (E) in (MPa)	Flow Stress (Yield Strength) in (MPa)	Ultimate Strength in (MPa)	Rupture Stress in (MPa)	Rupture Strain in (%)
BW30-22 °C (0.0001 s^−1^)	18.656	16.812	57.647	52.992	12.463
BW30-40 °C (0.0001 s^−1^)	17.692	11.856	49.859	49.229	13.978
BW30-22 °C (0.003 s^−1^)	19.718	17.175	62.217	62.005	12.264
BW30-40 °C (0.003 s^−1^)	20.365	15.817	52.382	49.314	13.259
BW30-22 °C (0.01 s^−1^)	18.670	13.663	53.802	53.018	12.330
BW30-40 °C (0.01 s^−1^)	18.418	13.613	51.012	50.867	12.275

## References

[1] WWAP (2018). The United Nations World Water Development Report 2018: Nature-Based Solutions for Water.

[2] Wada Y., Flörke M., Hanasaki N., Eisner S., Fischer G., Tramberend S., Satoh Y., van Vliet M.T.H., Yillia P., Ringler C. (2016). Modeling global water use for the 21st century: The Water Futures and Solutions (WFaS) initiative and its approaches. Geosci. Model Dev..

[3] Darre N.C., Toor G.S. (2018). Desalination of Water: A Review. Curr. Pollut. Rep..

[4] Miller D.J., Paul D.R., Freeman B.D. (2014). An improved method for surface modification of porous water purification membranes. Polymer.

[5] Szymczyk A., van der Bruggen B., Ulbricht M. (2019). Surface Modification of Water Purification Membranes. Surface Modification of Polymers: Methods and Applications.

[6] Miller D.J., Dreyer D.R., Bielawski C.W., Paul D.R., Freeman B.D. (2017). Surface Modification of Water Purification Membranes. Angew. Chem. Int. Ed..

[7] Kim S., Ou R., Hu Y., Li X., Zhang H., Simon G., Wang H. (2018). Non-swelling graphene oxide-polymer nanocomposite membrane for reverse osmosis desalination. J. Membr. Sci..

[8] Kamio E., Kurisu H., Takahashi T., Matsuoka A., Yoshioka T., Nakagawa K., Matsuyama H. (2021). Using Reverse Osmosis Membrane at High Temperature for Water Recovery and Regeneration from Thermo-Responsive Ionic Liquid-Based Draw Solution for Efficient Forward Osmosis. Membranes.

[9] Susanto H., Ulbricht M. (2009). Characteristics, performance and stability of polyethersulfone ultrafiltration membranes prepared by phase separation method using different macromolecular additives. J. Membr. Sci..

[10] Gholami S., Llacuna J.L., Vatanpour V., Dehqan A., Paziresh S., Cortina J.L. (2022). Impact of a new functionalization of multiwalled carbon nanotubes on antifouling and permeability of PVDF nanocomposite membranes for dye wastewater treatment. Chemosphere.

[11] Kang G.-D., Cao Y.-M. (2012). Development of antifouling reverse osmosis membranes for water treatment: A review. Water Res..

[12] Zhao S., Liao Z., Fane A., Li J., Tang C., Zheng C., Lin J., Kong L. (2021). Engineering antifouling reverse osmosis membranes: A review. Desalination.

[13] Wang K., Abdalla A.A., Khaleel M.A., Hilal N., Khraisheh M.K. (2017). Mechanical properties of water desalination and wastewater treatment membranes. Desalination.

[14] Aghajani M., Maruf S.H., Wang M., Yoshimura J., Pichorim G., Greenberg A., Ding Y. (2017). Relationship between permeation and deformation for porous membranes. J. Membr. Sci..

[15] Idarraga-Mora J.A., Ladner D.A., Husson S.M. (2018). Thin-film composite membranes on polyester woven mesh with variable opening size for pressure-retarded osmosis. J. Membr. Sci..

[16] Idarraga-Mora J.A., O’Neal A.D., Pfeiler M.E., Ladner D.A., Husson S.M. (2020). Effect of mechanical strain on the transport properties of thin-film composite membranes used in osmotic processes. J. Membr. Sci..

[17] Manawi Y.M., Wang K., Kochkodan V., Johnson D.J., Atieh M.A., Khraisheh M.K. (2018). Engineering the Surface and Mechanical Properties of Water Desalination Membranes Using Ultralong Carbon Nanotubes. Membranes.

[18] Warsinger D.M., Swaminathan J., Guillen-Burrieza E., Arafat H.A., Lienhard V.J.H. (2015). Scaling and fouling in membrane distillation for desalination applications: A review. Desalination.

[19] Alabtah F.G., Alkhouzaam A., Khraisheh M., Attia H. (2022). Characterization of surface properties of thin film composite (TFC) membranes under various loading conditions. CIRP Ann..

[20] Fard A.K., McKay G., Buekenhoudt A., Sulaiti H.A., Motmans F., Khraisheh M., Atieh M. (2018). Inorganic Membranes: Preparation and Application for Water Treatment and Desalination. Materials.

[21] Eziyi I., Krothapalli A., Osorio J.D., Ordonez J.C., Vargas J.V.C. Effects of Salinity and Feed Temperature on Permeate Flux of an Air Gap Membrane Distillation Unit for Sea Water Desalination. Proceedings of the 2013 1st IEEE Conference on Technologies for Sustainability (SusTech).

[22] Alkhouzaam A., Qiblawey H. (2021). Synergetic effects of dodecylamine-functionalized graphene oxide nanoparticles on antifouling and antibacterial properties of polysulfone ultrafiltration membranes. J. Water Process Eng..

[23] Alkhouzaam A., Qiblawey H. (2021). Functional GO-based membranes for water treatment and desalination: Fabrication methods, performance and advantages. A review. Chemosphere.

[24] Perera D., Song Q., Qiblawey H., Sivaniah E. (2015). Regulating the aqueous phase monomer balance for flux improvement in polyamide thin film composite membranes. J. Membr. Sci..

[25] Chauhan V.K., Singh J.P., Debnath S. (2020). Tensile behavior of virgin and recycled polyester nonwoven filter fabrics. J. Ind. Text..

[26] Pramanick A., Patra A., Ghosh R., Bhakta S. Studies on tensile properties of cross-laid nonwoven fabric. https://www.researchgate.net/publication/332548038_STUDIES_ON_TENSILE_PROPERTIES_OF_CROSS-LAID_NONWOVEN_FABRIC_Measurement_of_tensile_properties_in_cross_and_machine_direction_under_variable_gauge_length.

[27] Ray S.C., Ghosh P. (2017). Studies on Nature of Anisotropy of Tensile Properties and Fibre Orientation in Cross-Laid Needle-Punched Nonwoven Fabrics. Indian J. Fibre Text. Res..

[28] Le Clerc C., Bunsell A.R., Piant A. (2006). Influence of temperature on the mechanical behaviour of polyester fibres. J. Mater. Sci..

[29] Choi Y.J., Kim I., Kim S.H. (2019). Effect of heat-setting on the physical properties of chemically recycled polyester nonwoven fabrics. Text. Res. J..

